# The Story of the Insane from Year to Year

**Published:** 1892-05-14

**Authors:** 


### The Story of the Insane from Year to Year.

(Fourth Series.)

### ROYAL ASYLUM, PERTH.

The number of patients in this asylum rose from 93 to 101. There were 35 admissions, 8 recoveries, and 7 deaths. The recoveries stand at 22·85 per cent., and the deaths at 7 36. Alcoholism is the assigned of the insanity in three instances, and hereditary tendency accounts for 17. This institution has always been noted as one of the most admirably managed of its kind, and the history of the past year shows there has been no falling off, but rather that it has been advancing and extending its benefits. We learn that the average weekly cost per patient is £1 16s. 6d., and that, although the ordinary minimum rate of board is £60 per annum, 36 patients have been maintained at rates varying from £30 to £52. Amusement and recreation seem to be provided on the most liberal scale. Excursions were made to Edinburgh and other places, while there were 512 driving parties during the year, and 40 patients were taken to the seaside. The Commissioner in Lunacy says: "The asylum is under a very enlightened direction. A great deal has of late years been done to increase its efficiency, and all that has been done is admirable in its character."

### WARNEFORD HOUSE, OXFORD.

On January 1st, 1890, this asylum contained 81 patients, and at the end of the year the numbers had fallen to 76. Only 11 patients were admitted; five were discharged recovered, and seven died. Put into percentages in the usual manner we find the recoveries standing at 55·5, and the deaths at 8·8. In former notices we have drawn attention to the fact that this institution is one of the few of its class in England in which the charitable element is kept steadily in view. During the year many patients were kept at payments varying from 5s. a week to 15s. a week, and a few are put down at less than 3s. In all 68 were maintained at less than the actual cost. It would be well if every lunatic hospital in England were required to publish a similar table. The Commissioners in Lunacy say they "found the house in very good order, and there is much comfort throughout the building."

### THE ASYLUM FOR IDIOTS, REDHILL.

Not fewer than 599 patients were in residence on January 1st, 1890, and by the end of the year that number had risen to 622. The admissions were 82, the discharges 44, and the deaths 15. We learn from Dr. Jones' very interesting report that one-fourth of the cases admitted were suffering from epilepsy. We understand that some idiot asylums refuse to admit such cases at all, whatever may be their mental condition. Undoubtedly, they involve a great deal of extra trouble, and it is possible some harm may result to the better class patients from the constant presence of epileptics; but upon the whole we agree with Dr. Jones in thinking that they should be admitted in spite of the anxiety inseparable from their care and education. The Managers say they "are much supported by the able and scientific management of Dr. Jones, the Medical Superintendent, aided as he is by Mr. Cooper, the Assistant Medical Officer."

### BETHLEM HOSPITAL.

The numbers fell during the year from 249 to 229. There were 245 admissions, the recoveries were 127, and the deaths 28. The percentage of recoveries works out at 50 3 on the admissions, and the deaths at 11·8 on the average number resident. Dr. Smith's report runs to 23 pages, nine of them being taken up with a discussion on the new Lunacy Act, and we do not find a syllable in these nine pages in favour of the Act. Indeed, the more one reads of this Act the less one likes it, and the more one is filled with wonder as to who could have been responsible for it. When speaking of one provision of the Act, Dr. Smith says it "may compel the medical officers either to turn a dying person into the street or else to break the law." The clause about restraint prevented the use of padded gloves in a patient who, in about a fortnight, tore up "24 strong quilted sleeping rugs, each containing a blanket, and nine strong quilted and flannel-lined dresses, the whole costing the hospital close upon £50." Parallel cases are in the experience of almost every Medical Superintendent.

### WONFORD HOUSE, EXETER.

This lunatic hospital contained 124 patients at the close of the year, being an increase of 7 above the number on January 1st. The admissions were 35, the recoveries 14, and the deaths 5. These numbers show the percentage of recoveries to be 51·8, and the deaths 4. Drink is credited with the causation of insanity in four cases, hereditary tendency in 9, and influenza in 3. Dr. Deas points out that this "mysterious disease had in most cases a powerful specific effect on the nervous system." Dr. Deas has his shot at the new Lunacy Act, which he declares to be "retrograde in principle, and most irritatingly complex and cumbersome." And he ends his report in the following words: "The more one realises the spirit of the Act, and the more one sees of it, the less one likes it, and it is sincerely to be hoped that before many years have passed some of the more objectionable provisions will be modified." The managers say that the hospital is fulfilling well the object for which it was established; but they also say that only 20 patients out of a total of 124 were paying 20s. a week, or less.

### DEVON COUNTY ASYLUM.

The numbers increased during the year from 923 to 968. There were 227 admissions, 71 recoveries, and 91 deaths. The recoveries stand at 31·71 on the admissions, and the deaths at 9·66 on the average number resident. Hereditary influence accounts for 72 of the admissions, and drink for 19, so that had the inhabitants of Devonshire led proper lives, and with a higher sense of their responsibilities, the admissions to the County Asylum would only have been 136 instead of 227, and this has been going on as far back as statistics reach. No lesson has been learnt by the people—no crusade against these evil warnings and evil drink has been preached. There is matter enough in the reports of our county asylums to give point to a thousand homilies, and yet they hardly receive a glance from the periodical press. Dr. Saunders says, "The Local Government Act of 1888, and the Lunacy Act of 1890, have materially altered the old *régime*, and cast an unknown burthen of extra clerical and other work on the Medical Superintendent and other officers. It is doubtful how far this is for the benefit of the patients." Dr. Saunders may rest assured that no benefit, but quite the opposite, results to the patient—and to everyone else concerned.

---

It is almost too much to hope that a curative treatment has really been found for hydrophobia. One experiment, however successful, is insufficient basis on which to form a judgment, and the subcutaneous injection of the virus in its fixed form, which is stated to have been performed by Professor Murri, of Milan, with such conspicuous success on a man who had undergone the Pasteur treatment without avail must be repeated often under varying conditions before it can be pronounced a specific.

